# Conjunctival Melanoma: A Case Presentation

**DOI:** 10.30699/IJP.2023.557981.2941

**Published:** 2023-10-15

**Authors:** Fatemeh Montazer, Seyed Mohammad Heshmati, Salar Asgari, Shabnam Mollazadehghomi

**Affiliations:** Department of Pathology, School of *Medicine*, Iran University of Medical Sciences, Tehran, Iran

**Keywords:** Conjunctiva, Melanoma, Nevus, Neoplasms

## Abstract

Conjunctival melanoma is an uncommon tumor that is likely to recur and carries an overall mortality rate of approximately 30%. Melanoma arises from melanocytes, most often in sun-exposed skin. Less commonly, melanoma originates from other tissues such as the uvea, rectum, mouth, respiratory tract, and conjunctiva. Conjunctival melanoma represents only 1.6% of all noncutaneous melanoma. Herein, we reported a case of conjunctival melanoma followed by reviewing the literature to provide an optimal diagnostic approach.

## Introduction

Both benign and malignant lesions can appear on the conjunctiva. According to the presence of pigmentation as well as histopathologic involvement of melanocytes or its lack, these lesions can be categorized as melanocytic or non-melanocytic ones (1). Non-melanocytic lesions can be subdivided into epithelial, vascular, lymphoid, fibrotic, and metastatic types, while melanocytic ones are mainly nevus in more than a half of lesions followed by melanoma with malignant features and acquired melanosis as an atypical pigmented lesion (2). Such lesions can commonly appear on the bulbar conjunctiva but can be rarely found on the eyelid margin or caruncle (3). One of the extremely rare melanocytic lesions is conjunctival melanoma.

Malignant melanoma emerges from melanocytes, commonly in parts of skin that are sun-exposed. It is less common to observe melanoma in other tissues such as the uvea, rectum, mouth, respiratory tract, and conjunctiva. Conjunctival melanoma constitutes only 1.6% of all noncutaneous melanoma (4). Herein, we reported a case of conjunctival melanoma followed by reviewing the literature to provide an optimal diagnostic approach.

## Case Presentation

**Fig. 1:**
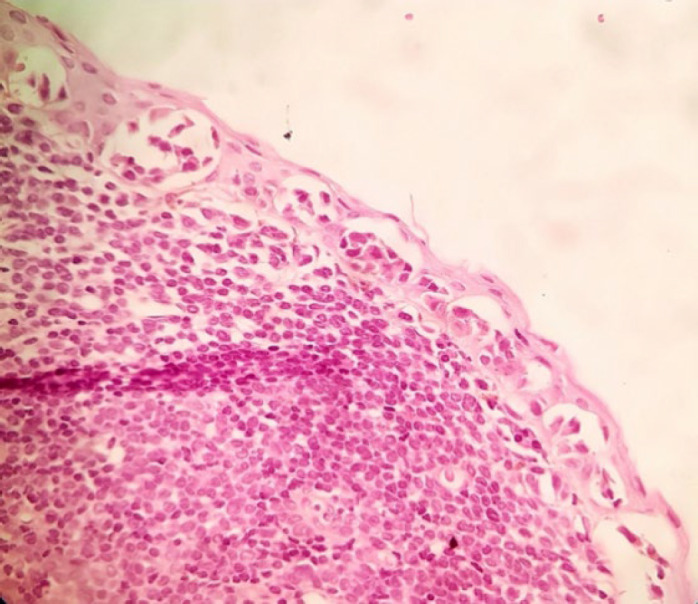
H&E×100: Partial replacement of the epithelium by atypical melanocytes with irregular hyperchromatic nuclei

**Fig. 2:**
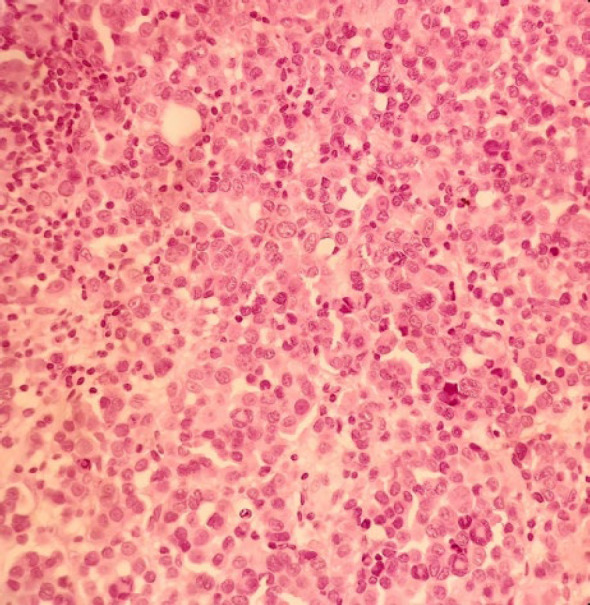
H&E×200:Large atypical tumoral cells with nuclear pleomorphism, abundant cytoplasm, and prominent nucleoli

**Fig. 3:**
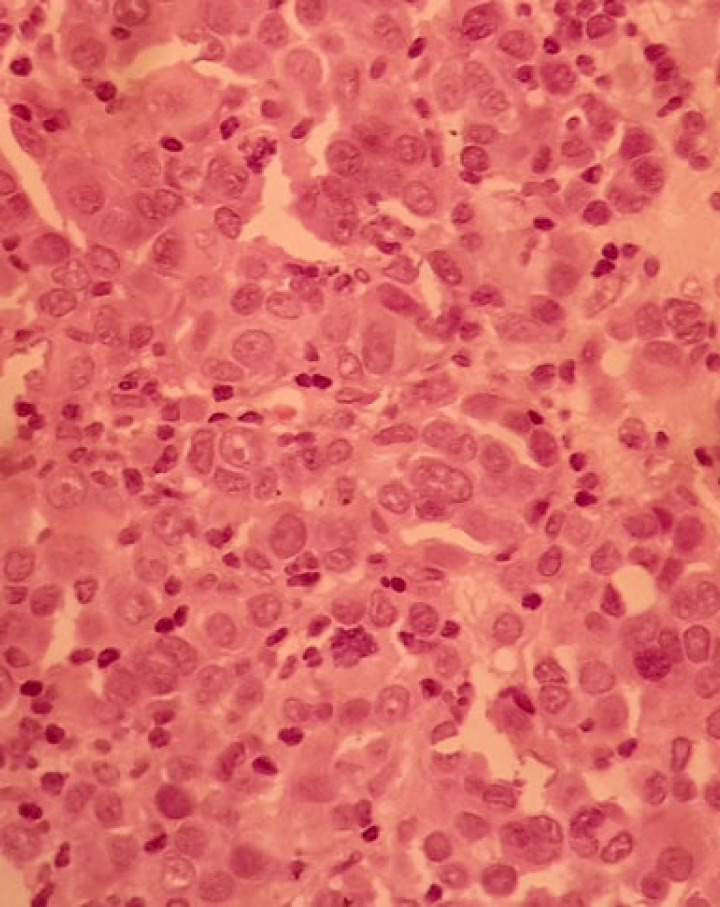
H&E×400: Nuclear pleomorphism with frequent mitoses in the deep component

**Fig. 4:**
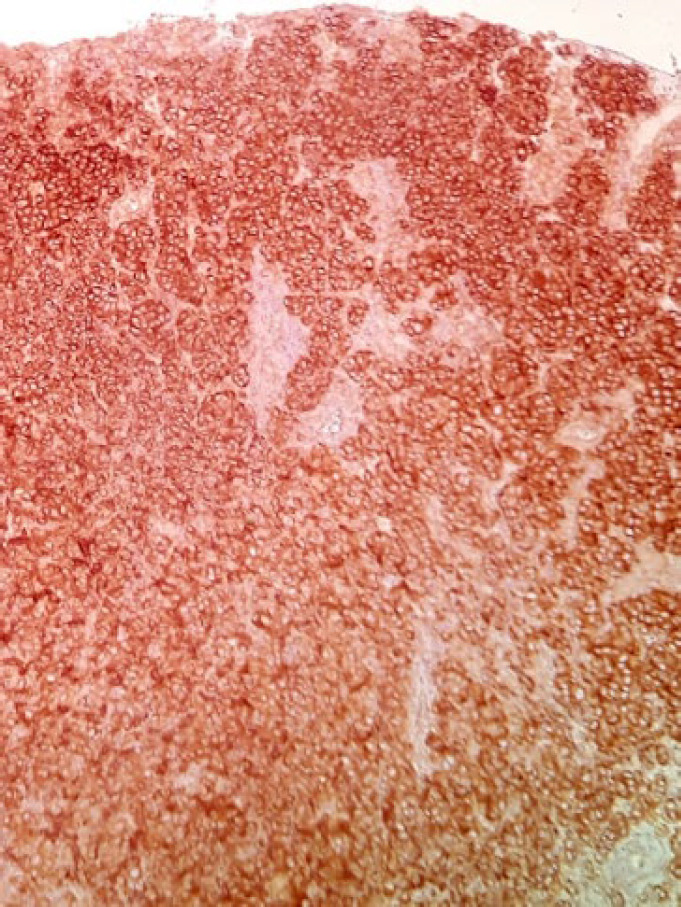
IHC×200: A strong positivity for Melan-A throughout the tumor

**Fig. 5:**
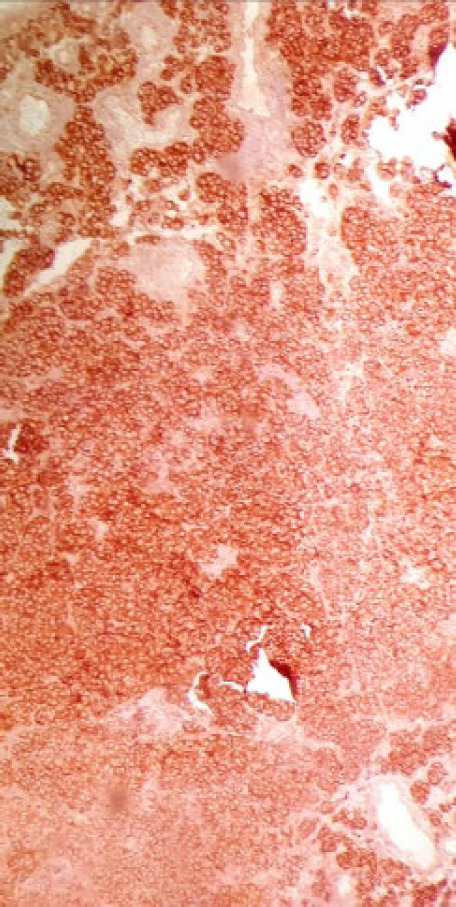
IHC×200: a strong positivity for HMB45 throughout the tumor

**Fig. 6:**
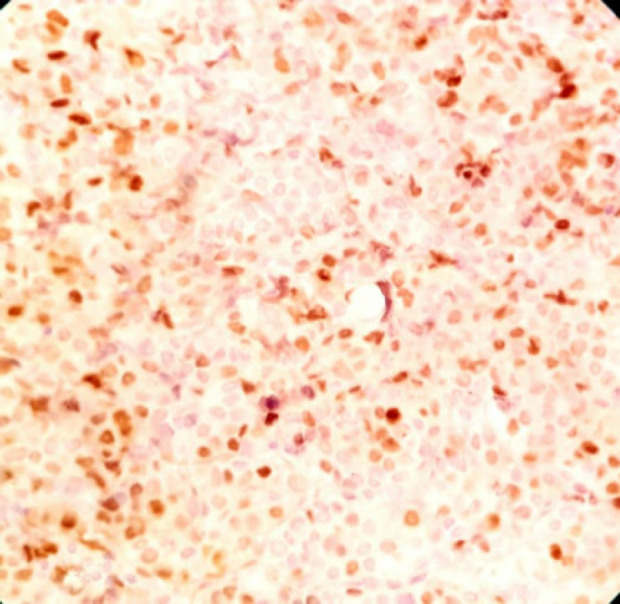
IHC×400: Ki67 stain shows a high proliferation index, located in superficial and deep aspects of the subepithelial component

The patient was a 34-year-old female with a history of a pigmented spot on the conjunctiva from childhood. This spot had not changed over the years, but in the last two months, the lesion had become prominent and polypoid and its size reached about one centimeter. The lesion was completely painless surrounded by erythematous borders. No history of underlying disease was reported. A biopsy was taken from the lesion. On histological assessment, a nodular melanocytic neoplasm composed of variable-sized intraepithelial nests of melanocytes with occasionally pagetoid growth patterns as well as a surface epithelial ulcer was evident (Figure 1). Assessment of the subepithelial components showed proliferation of uniform atypical melanocytes mostly with round epithelioid appearance and visible to prominent nucleoli (Figure 2). Scattered mitosis (in about 3 per 10 HPF) was also present in superficial and deep components (Figure 3). Infiltration of lymphocytes at the lateral and baseline of the lesion was found. Furthermore, cellular maturation at the base of the lesion was not identified. No melanin pigment was seen. Binucleated and multinucleated melanocytes were also observed and the tumor was completely vascular. The histological feature was consistent with conjunctival melanoma. For more confirmatuion and better assessment of the lesion, an immunohistochemical (IHC) study using for the HMB-45, Melan A, Ki67, Cyclin D1, P53, and bcl2 stains was recommended which showed positivity for Melan A in most of the tumoral cells with diffuse pattern in superficial and deep components (Full thickness) (Figure 4), HMB-45 positivity in tumor cells with patchy distribution in superficial and deep components (Figure 5), bcl2 with moderately to strongly positivity in most tumor cells in superficial and deep components, Cyclin D1 positivity in some tumor cells with patchy distribution in superficial and deep components. Ki67 labeling index was noted in about 10% of the tumoral cells in superficial and deep components (Figure 6). According to such immunolabeling, a final diagnosis of conjunctival melanoma was made.

## Discussion

Malignant melanoma is a potentially fatal tumor that arise from melanocytes and commonly seen in sun-exposed skin. Melanoma is rarely reported in other tissues such as the uvea and mucous membranes, including the vulva, rectum, mouth, respiratory tract, and conjunctiva. Conjunctival melanoma accounts only 1.6% of all noncutaneous melanoma (4) and is likely to recur, carrying an overall mortality rate of approximately 30% (5).

Two clinical and pathologic forms of conjunctival melanoma are reported; tumors evolving indirectly following a variably protracted course of primary acquired melanosis (PAM) (termed melanoma with PAM) and the ones that evolve directly without antecedent PAM (melanoma without PAM). A likely serious melanocytic lesion is the PAM of the con-junctiva, which might results in melanoma development (6).

Of conjunctival melanomas, roughly 75% would emerge in the context of antecedent PAM (7). 

When PAM is inapparent, a short intraepithelial growth phase is observed while a vertically invasive phase quickly follows. In around 20% to 30% of the cases with conjunctival melanomas (with or without PAM) histologic evidence of a preexistent nevus or a history of such lesion from childhood are reported (8,9).

Histopathologic features are considered as important prognostic factors. Increased tumor thickness in conjunctival melanoma is associated with an increased risk of regional nodal and distant metastasis (10). Therefore, in conjunctival melanomas with less than 2 mm thickness, regional nodal metastases are rare (11). Conjunctival melanoma in association with ulceration carries an increased risk for regional lymph node metastasis (12).

It is important to distinguish conjunctival melanoma from benign nevi lesions. Conjunctival Spitz nevus was primarily described when some patients were reported with benign juvenile melanoma, in whom histological features of malignant lesions were present without prominent malignant behavior (12).

Some of the authors proposed that cytological atypia might not always be equivalent to malignancy, but in some cases, they described that fatal metastasis was also revealed. These findings may lead to a major diagnostic challenge among pathologists (12). How-ever, through developing some specific antibodies and assessment of their expression, atypical conjunctival Spitz nevi from other conjunctival lesions may be differentiated. 

Despite the prevalence of conjunctival nevi as a benign phenomenon, few cases of atypical conjunctival Spitz nevus were reported with malignant behaviors. In the latter lesions, the prominent feature was nevic cell proliferation forms as nests along epithelial and subepithelial junctions (13). In some cases, especially at higher ages, conjunctival nevi may undergo progressive maturation causing conjunctival mela-noma. In some cases, the differential diagnosis of such tumors from acquired melanosis may be difficult (12). As a major concern, differential diagnosis of Spitz nevus from malignant melanoma is very important. In this regard, using histological features along with antibody labeling should be considered. Immuno-histochemical staining of common cutaneous nevome-lanocytic nevi and melanomas with antibodies directed against S-100, HMB-45, and MART-1 antigens have yielded valuable diagnostic data (14). 

The appearance of conjunctival melanocytic lesions among children or young adults, along with some histological features including variation in pigmen-tation, symmetry, sharp lateral borders, and cellular maturation with negative, weak, or superficial staining for melanoma-related markers, especially S100, MIB-1, HMB-45, and Melan A (MART-1) seen in Spitz nevus versus common malignant behaviors, including recurrence or metastasis as well as expression of HMB-45 along with strong immunoreactivity for MIB-1 observed in in melanoma, are counted as useful distinguishing features (12, 15, 16).

Despite the provision of various methods and tools for the diagnosis of these lesions, similarly described cases have been associated with a variety of histo-logical and clinical behaviors. In a study by Colarossi *et al.* in 2013 (17), the atypical conjunctival nevi were mainly characterized by cystic dilatation of the con-junctival gland, lack of mitotic activity, marked melanocytic atypia and severe inflammation, with IHC positivity for Melan-A, HMB-45, and Ki-67 antibodies. In the case of Morkin *et al.* (18) in 2018, conjunctival nevi extending onto the cornea, mimi-cking a pterygium was described. The lesions had reverse maturation but without mitotic figures. In the assessment of immunoactivity, diffuse positivity for Mart-1, S100, SOX-10, and superficial immuno-reactivity to MITF and HMB-45 were described. In a conjunctival spitz nevus case reported by Vervaet *et al.* (19), the histological assessment showed nests of large, polygonal, non-pigmented epithelioid cells, which demonstrated immunoreactivity for S-100 protein and tyrosinase-associated protein; as well as a superfiacial immunoreactivity for HMB45 and MIB-1.

## Conclusion

Considering the rarity of conjunctival melanoma and its very high mortality rate, observation of a melanocytic lesion in the conjunctiva, especially a rapidly growing one, as was shown in our case , should be given special attention to rule out malignant melanoma of the conjunctiva.

## Conflict of Interest

The authors declared no conflict of interest.
